# Prevalence and Risk Factors for HTLV-1/2 Infection in Quilombo Remnant Communities Living in the Brazilian Amazon

**DOI:** 10.3389/fpubh.2022.871865

**Published:** 2022-03-30

**Authors:** Wandrey Roberto dos Santos Brito, Greice de Lemos Cardoso-Costa, Lourival Marques Roland Junior, Keise Adrielle Santos Pereira, Felipe Teixeira Lopes, Bernardo Cintra dos Santos, Aline Cecy Rocha de Lima, Isabella Nogueira Abreu, Carlos Neandro Cordeiro Lima, Sandra Souza Lima, Izaura M. Vieira Cayres Vallinoto, Eduardo José Melo dos Santos, João Farias Guerreiro, Antonio Carlos Rosário Vallinoto

**Affiliations:** ^1^Laboratory of Virology, Institute of Biological Sciences, Federal University of Pará, Belém, Brazil; ^2^Postgraduate Program in Biology of Infectious and Parasitic Agents, Institute of Biological Sciences, Federal University of Pará, Belém, Brazil; ^3^Laboratory of Human and Medical Genetics, Institute of Biological Sciences, Federal University of Pará, Belém, Brazil; ^4^Laboratory of Genetics of Complex Diseases, Institute of Biological Sciences, Federal University of Pará, Belém, Brazil

**Keywords:** HTLV-1/2, Amazon, quilombos, epidemiology, vulnerable population

## Abstract

Human T-lymphotropic viruses 1 and 2 (HTLV-1 and HTLV-2) are retroviruses that originated on the African continent and dispersed throughout other continents through human migratory flows. This study describes the prevalence of HTLV-1 and HTLV-2 infection in residents of 11 quilombo remnant communities in the state of Pará, Brazil, and the associated risk factors. A total of 859 individuals (334 men and 525 women), aged between 7 and 91 years, participated in the study. All subjects answered a questionnaire with questions on sociodemographic characteristics and on risk factors associated with HTLV infection, and blood samples were collected and separated into plasma and leukocytes. An immunoenzymatic assay (ELISA; Murex HTLV-I+II, DiaSorin, Dartford, UK) was used as a screening test, and positive samples were subjected to line immunoassay confirmatory tests (Inno-LIA HTLV I/II Score FUJIREBIO) and DNA extraction for subsequent real-time PCR to differentiate the viral type. Four of the 859 individuals were seropositive for HTLV. HTLV-1 infection was confirmed in one individual from the Itamoari community (0.92%), and HTLV-2 infection was confirmed in two individuals from São Benedito (3.17%) and in one individual from Arimandeua (2.22%). Blood transfusion was the only risk factor associated with HTLV infection in this study. This study reports the occurrence of HTLV-1 and HTLV-2 in quilombo remnant communities in the state of Pará. Considering the African origin of the virus and its introduction into Brazil from the slave trade, the continued evaluation of quilombola communities in the state of Pará is essential to better characterize the distribution of infections in these populations and to create public health policies for the control of the spread of the virus and associated diseases.

## Introduction

Human T-lymphotropic viruses 1 (HTLV-1) and 2 (HTLV-2) were the first isolated retroviruses to infect humans (1, 2). They are viruses with known tropism for T lymphocytes and belong to the family *Retroviridae*, subfamily *Orthoretrovirinae*, and genus *Deltaretrovirus* (3–5). Evidence suggests an African origin for both viruses from independent events of interspecies transmission of the simian T-lymphotropic virus (STLV-1 and STLV-2) from non-human primates to humans (6–8). HTLV-1 has been associated with several diseases, particularly a neoplasm, i.e., adult T cell leukemia/lymphoma (ATLL), and an inflammatory neurodegenerative disease, i.e., HTLV-1-associated myelopathy (HAM) (9–11). In addition, HTLV-1 is also associated with ocular (uveitis) (12, 13), rheumatologic (14) and pulmonary (15, 16) diseases, among other pathologies (17–19). HTLV-2, in turn, does not have clinical manifestations necessarily associated with infection; however, clinical symptoms have been described in some patients (20, 21).

The main forms of HTLV-1/2 transmission are unprotected sex (22, 23), blood transfusion (24), sharing syringes and needles (25), and vertical transmission, in particular, prolonged breastfeeding (26, 27). HTLV-1 is widely distributed in geographic areas such as sub-Saharan Africa, Japan, Melanesia, the Caribbean, and South America (28–31). HTLV-2 has a more restricted distribution to pygmy populations in Africa and to indigenous peoples of the Americas, especially in the Brazilian Amazon region (7, 8, 32–36).

The entry of HTLV-1 into Brazil must have occurred along the east coast of the country, between the 16th and 19th centuries, through the African slave trade (37, 38). HTLV-2 may have followed the oldest human migratory flows that occurred thousands of years ago from the Asian continent via the Bering Strait, reaching North America and moving toward South America and, thus, Brazil (35, 37, 39). In Brazil, the viruses are distributed throughout the territory, and the main foci of infection are found in the North and Northeast regions (40). In the North region of the country, in the state of Pará, HTLV-1, and HTLV-2 have been described in several population strata, including urban, rural, and riverine populations, blood donors, and pregnant women, in addition to the presence of coinfections in people living with HIV (PLHIV), intravenous drug users, and isolated populations such as indigenous and quilombola peoples (25, 41–47). During the colonial period in Brazil, enslaved blacks in search of freedom fled coffee and sugarcane farms to the forest interior, where they formed isolated societies called quilombos. In the state of Pará, there are indications that quilombos formed in the 18th century, of which many were formed on the banks of rivers, such as the Gurupi, Tocantins, and Trombetas rivers (48). Many quilombo remnant communities lack basic sanitation, which promotes the transmission of diseases and fosters precarious health conditions; thus, this is one of the most vulnerable populations in Brazil (49–51). Infections by HTLV-1 and HTLV-2 have already been described in quilombo remnant communities in the Marajó Archipelago (43), but there are no studies on these viruses in other quilombos in the state of Pará. Thus, the aim of this study was to describe the prevalence of HTLV-1 and HTLV-2 and the main risk factors in 11 quilombola communities in the state of Pará.

## Materials and Methods

### Study Population and Sample Collection

**Figure 1:**
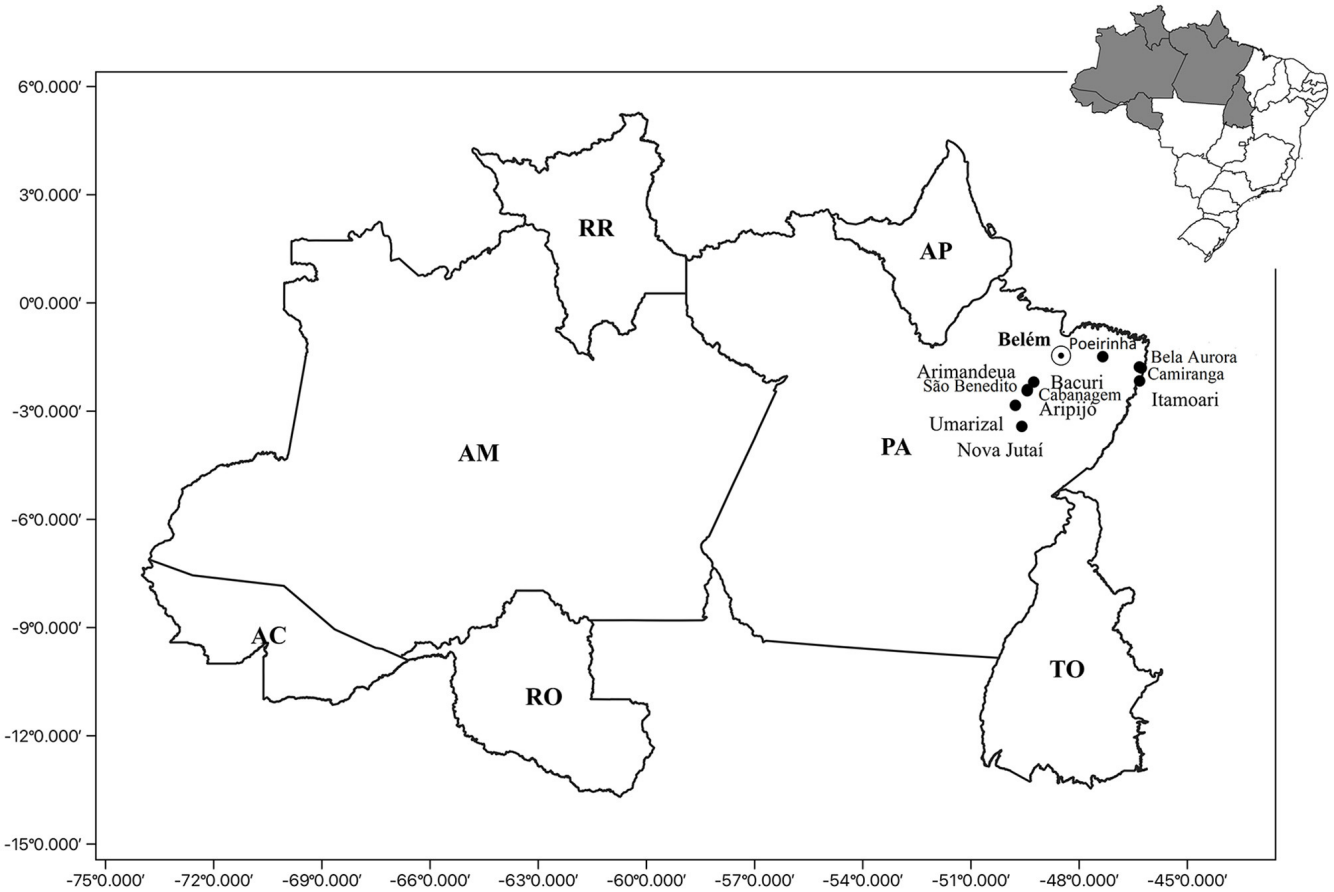
Geographic location of the quilombolas communities: Poeirinha, municipality of Bonito; Bela Aurora, Camiranga and Itamoari, municipality of Cachoeira do Piriá; Arimandeua, Aripijó Bacuri, Cabanagem, and São Benedito, municipality of Cametá; Umarizal Beira, municipality of Baião; and Nova Jutaí, municipality of Breu Branco. PA, Para State; Belém, capital of the Para State; AP, Amapá State; TO, Tocantins State; RR, Roramia State; AM, Amazonas State; AC, Acre State; RO, Rondônia State.

A total of 11 quilombola communities located in the state of Pará (northern Brazil) were visited from September 2020 to April 2021, and information was collected from 859 individuals. The following communities were studied: Poeirinha (*n* = 20), Umarizal Beira (*n* = 303), Arimandeua (*n* = 45), Aripijó (*n* = 31), Bacuri (*n* = 10), Cabanagem (*n* = 17), São Benedito (*n* = 63), Bela Aurora (*n* = 35), Camiranga (*n* = 89), Itamoari (*n* = 109), and Nova Jutaí (*n* = 137) (Figure 1).

All study participants signed an informed consent form and answered a questionnaire with questions about sociodemographic aspects (age, sex, marital status, education, and family income) and behavioral risk factors for exposure to HTLV (“tattoos,” “use of illicit drugs,” “blood transfusion,” “piercings,” “sexually active,” “use of condoms,” and “previous diagnosis of STI,” among others). Individuals under 18 years old signed an informed assent form, and their legal guardians signed an informed consent form.

From all participants, peripheral blood samples (4 mL) were collected in vacuum tubes containing ethylenediaminetetraacetic acid (EDTA) as an anticoagulant. The samples were then subjected to centrifugation to separate the plasma and leukocyte fractions, followed by storage at −20°C until use.

### Ethical Aspects

This study was approved by the National Research Ethics Committee (CONEP, acronym in Portuguese) (CAAE: 27290619.2.0000.0018), in compliance with resolution 466/12 of the Ministry of Health, which regulates research involving human beings. All subjects who voluntarily agreed to participate in the study signed an informed consent form.

### Serological Analysis

Immunoenzymatic assays (Murex HTLV-I+II, DiaSorin, Dartford, UK) were used to detect anti-HTLV-1/2 antibodies in the tested samples following the manufacturer's instructions. Samples with positive results were subjected to line immunoassays (INNO-LIA^®^ HTLV I/II Score, Fujirebio, Japan) and real-time PCR (Applied Biosystems Step One Plus Real Time PCR) to confirm infection and differentiate the viral type, respectively.

### Real-Time PCR

To perform real-time PCR, DNA was extracted from the seropositive samples using a *QiaAmp* DNA mini kit (Qiagen, Germany) following the manufacturer's protocol. Molecular confirmation of infection was performed using a multiplex real-time PCR for three target sequences: the albumin gene (141 bp), as an endogenous control; the *pol* gene (186 bp) of HTLV-1; and the *tax*-2 gene (75 bp) of HTLV-2. The primer sequences used were 5′-ccctacaatccaaccagctcag-3′ (HTLV-1F), 5′-gtggtgaagctgccatcgggtttt-3′ (HTLV-1R), 5′-cgattgtgtacaggccgattg-3′ (HTLV-2F), 5′-caggagggcatgtcgatgtag-3′ (HTLV-2R), 5′-gctgtcatctcttgtgggctgt-3′ (F Albumin), 5′-aaactcatgggagctgctggtt-3′ (R Albumin); the probe sequences used were JOE-5′-ctttactgacaaacccgacctacccatgga-3′-BHQ (HTLV-1), FAM-5′-tgtcccgtctcaggtggtctatgttcca-3′-MGB (HTLV-2), and NED-5′-cctgtcatgcccacacaaatctc-3′-MGB (albumin). The reaction components, based on the *TaqMan*^®^ Universal system, were as follows: 12.5 μL of Master Mix, 6.0 μL of water, 1.0 μL of Assay-by-Design (primer and probe set) and 0.5 μL of DNA in a final volume of 20 μL. For cycling, the protocol was as follows: 50°C for 2 min; 95°C for 10 min; and 50 cycles of 90°C for 50 s and 60°C for 1 min.

### Statistical Analyses

The collected information was stored in a database using Epi-Info7.2 software. The socioeconomic aspects, behavioral factors, and risk factors for exposure to HTLV-1 and HTLV-2 are described as frequencies and percentages. The confidence interval (95% CI) was used to estimate overall and community prevalence. Fisher's exact test was used to evaluate the association of behavioral risk factors with the presence/absence of HTLV-1 or HTLV-2. The significance level adopted was 5%. Statistical analyses were performed using the programs BioEstat 5.3, GraphPad Prism 7.0 for Windows, and MINITAB Release 14 for Windows.

## Results

The median age of the individuals was 39 years (IIQ: 32); 61.1% (*n* = 525) of the individuals were women, and 38.9% (*n* = 334) were men. A total of 49.9% (*n* = 429) of the individuals said they were married/living with a partner. Most of these individuals declared themselves black (60.5%; *n* = 520) and brown (33.5%; *n* = 288). Regarding education, 40.6% (*n* = 349) were literate, 16.5% (142) had completed elementary school, and 28.4% (*n* = 244) had completed secondary education. A total of 46.6% (*n* = 400) live on <1 (1) minimum wage, and 47.1% (*n* = 405) live on one (2) to three (3) minimum wages (Table 1).

**Table 1:** Sociodemographic profile of the study participants.

**Sociodemographic variable**	***N*** **= 859**	**%**
**Sex**		
Female	525	61.1
Male	334	38.9
**Color**		
Yellow	9	1.0
White	42	4.9
Black	520	60.5
Brown	288	33.5
**Marital status**		
Married/living with partner	429	49.9
Separated	59	6.9
Single	311	36.2
Widowed	41	4.8
Not reported	19	2.2
**Education**		
Illiterate	22	2.6
Literate	349	40.6
Elementary education	142	16.5
Secondary education	244	28.4
Higher education	63	7.3
Graduate	15	1.7
Not reported	24	2.8
**Income**		
<1	400	46.6
1–3	405	47.1
>3	7	0.8
Not reported	47	5.5
**Age (years)**		
07–11	45	5.2
12–18	108	12.6
19–29	141	16.4
30–59	424	49.4
60 or 60+	136	15.8
Not reported	5	0.6

HTLV-1/2 infection was detected in four individuals (0.4%, 95% CI: 0.13–1.19). Table 2 provides the distribution of infection by community, with 3.2% (2/63) of those tested in São Benedito (95% CI: 0.38–11) and 2.2% (1/45) of those tested in Arimandeua (95% CI: 0.05–11) being positive for HTLV-2 infection and 0.9% (1/109) of those tested in Itamoari (95% CI: 0.02–5.00) being positive for HTLV-1 infection; all of the communities are located in the northeastern region of the state of Pará. HTLV-1 infection was identified in a young, 24-year-old male. Two males and one female were diagnosed with HTLV-2; all three were over 60 years of age.

**Table 2:** Prevalence of HTLV-1 and HTLV-2 in the communities based on the tests used.

**Population**	***N***	**ELISA**	**Inno-Lia**		**Real-time PCR**	
			**HTLV-1 (%)**	**HTLV-2 (%)**	**HTLV-1 (%)**	**HTLV-2 (%)**
**Baião**						
Umarizal Beira	303	00	–	–	–	–
**Breu Branco**
Nova Jutaí	137	00	–	–	–	–
**Bonito**						
Poeirinha	20	00	–	–	–	–
**Cachoeira do Piriá**						
Bela Aurora	35	00	–	–	–	–
Camiranga	89	00	–	–	–	–
Itamoari	109	01	01 (0.9)	–	01 (0.9)	–
**Cametá**						
Arimandeua	45	01	–	01 (2.2)	–	01 (2.2)
Aripijó	31	00	–	–	–	–
Bacuri	10	00	–	–	–	–
Cabanagem	17	00	–	–	–	–
São Benedito	63	02	–	02 (3.2)	–	02 (3.2)
**Total**	859	04	01 (0.1)	03 (0.3)	01 (0.1)	03 (0.3)

Only the individual diagnosed with HTLV-1 infection reported having a tattoo (25%). Three individuals (75%) denied having used illicit drugs, and one did not report anything. All of them denied having piercings, a previous diagnosis of an STI, or having more than one sexual partner. Two (50%) of those infected reported having received blood transfusions. Half (2) of the diagnosed individuals were sexually active and used condoms during sexual intercourse. Three (75%) of the individuals were breastfed, and one individual did not know (Table 3). According to Fisher's exact test, there was a significant association between blood transfusion (*p* = 0.0176) and HTLV-1/2 infection, with no association with any of the other factors investigated (Table 4).

**Table 3:** Sociodemographic and behavioral characteristics of individuals with confirmed HTLV-1 and HTLV-2 infection.

**Characteristics**	**Infected individuals**			
	**SBEN-002**	**SBEN-019**	**ARI-007B**	**ITM-040**
Sex	F	M	M	M
Age	64	67	67	23
Color	Black	Black	Brown	Brown
Marital status	Single	Single	Married/lives with partner	Single
Education	IE	IE	IE	IE
Income (number of minimum wages)	1	>1	1	>1
Tattoo(s)	N	N	N	Y
Piercing(s)	N	N	N	N
Blood transfusion	Y	N	N	Y
Breastfed	Y	NR	Y	Y
Sexually active	N	N	Y	Y
Age at 1st sexual relationship	NR	NR	18	NR
Sex for money	N	N	N	NR
Condoms	N	N	Y	Y
Number of partners	NR	1	1	1
Diagnosis of an STI	N	N	N	N
Type of HTLV	HTLV-2	HTLV-2	HTLV-2	HTLV-1

*F, female; M, male; IE, incomplete elementary school; IM, incomplete middle school; N, no; Y, yes; NR, not reported*.

**Table 4:** Association of HTLV-1/2 infection with behavioral risk factors.

	**Positive**	**Negative**	**Grand total**	***P***
	***n*** **= 4**	***n*** **= 855**	***n*** **= 859**	
	***N*** **(%)**	***N*** **(%)**	***N*** **(%)**	
**Tattoo(s)**				
Yes	1 (25)	51 (6)	52 (6.1)	
No	3 (75)	778 (91)	781 (90.9)	0.2276
Not reported		26 (3)	26 (3)	
**Illicit drugs**				
Yes	0 (0)	34 (4)	34 (4)	
No	3 (75)	756 (88.4)	759 (88.4)	1.000
Not reported	1 (25)	65 (7.6)	66 (7.7)	
**Blood transfusion**				
Yes	2 (50)	44 (5.1)	46 (5.4)	
No	2 (50)	763 (89.2)	765 (89.1)	0.0176
Not informed		48 (5.6)	48 (5.6)	
**Piercing(s)**				
Yes	0 (0)	21 (2.5)	21 (2.4)	
No	4 (100)	781 (91.3)	785 (91.4)	1.000
Not reported		53 (6.2)	53 (6.2)	
**Breastfed**				
Yes	3 (75)	774 (90.5)	777 (90.5)	
No	0 (0)	27 (3.2)	27 (3.1)	1.000
Not reported	1 (25)	54 (6.3)	55 (6.4)	
**Sexually active**				
Yes	2 (50)	637 (78.6)	639 (78.5)	
No	2 (50)	159 (19.6)	161 (19.8)	0.1823
Not reported	0 (0)	14 (1.7)	14 (1.7)	
**Number of sexual partners (week)**				
1	2 (100)	532 (83.5)	534 (83.6)	
≥2	0 (0)	23 (3.6)	23 (3.6)	0.1000
Not reported		82 (2.9)	82 (2.8)	
**Condoms**				
Yes	2 (50)	262 (32.3)	264 (32.4)	
No	2 (50)	459 (56.7)	461 (56.6)	0.6279
Not reported	0 (0)	89 (11)	89 (10.9)	
**Diagnosis of an STI**				
Yes	0 (0)	32 (3.7)	32 (3.7)	
No	4 (100)	677 (79.2)	681 (79.3)	1.000
Does not know	0 (0)	34 (4)	34 (4)	
Not reported	0 (0)	112 (13.1)	112 (13)	

## Discussion

Herein, we report the occurrence of anti-HTLV-1/2 antibodies in four of the 859 individuals tested, with confirmed HTLV-1 infection in only one resident of the Itamoari community and confirmed HTLV-2 infection in two communities—Arimandeua and São Benedito. This is the first time that HTLV-1 and HTLV-2 have been identified in these communities, and no previous studies have been conducted in these communities.

Vallinoto et al. (43) found that the prevalence rate of HTLV-1 in quilombolas of the Marajó Archipelago (Pará) ranged from 1.0% in the Ponta de Pedras community to 2.06% in Santana do Arari; these numbers are close to those found in this study. Regarding the prevalence of HTLV-2, the rates found in this study were also similar to those described in Santana do Arari (1.06%). Regarding other Afro-descendant populations studied in Brazil, the prevalence of HTLV-1 in the present study was similar to that found in quilombo remnant communities in Central Brazil (0.5%) (52).

The prevalence of HTLV-2 in the present study was lower than that reported by other studies involving traditional populations of Pará, such as indigenous populations. The prevalence in three Kayapó villages in the Xikrin territory was 29%, ranging from 21 to 38% among the villages (41). In turn, the prevalence data for this study are consistent with the data for the population of Belém, the capital of Pará, and for blood donors in this same state (42, 44).

Itamoari is a community located on the Pará side of the Gurupi River. The Gurupi River and the Turiaçu River (State of Maranhão) form a region called Turiaçu. The port in this region served as an entrance for a large number of slave through illegal slave trade during the 19th century (48). In this sense, the occurrence of HTLV-1 in the Itamoari community may be related to the African origin of the virus, and its entry into Brazil may have been by the slave trade from the African continent, considering that cases occur in isolated communities.

There are historical reports (48) that slaves sometimes found indigenous tribes during their escape into forests and lived with residents in locations highly endemic for HTLV-2 (48). Thus, the detection of HTLV-2 in São Benedito and Arimandeua can be attributed to the contact between indigenous populations and slaves.

History of an STI, multiple sexual partners, and non-use of condoms are risk factors that have been associated with a higher risk of HTLV-1/2 infection; however, in the present study, no associations were found (23, 25, 53). Nevertheless, two individuals infected with HTLV-2 reported not using condoms because they were no longer sexually active, leading to the hypothesis or inference that they did not use condoms in previous relationships. None of the four infected had any history of an STI or reported having more than one sexual partner.

Blood transfusion was a risk factor for HTLV-1/2 infection in this study as well as in other studies (54, 55). In November 1993, the Brazilian Ministry of Health filed Ordinance No. 1,376, through which blood donation candidates are subjected to clinical screening, including HTLV screening, to ensure their safety and that of recipients (56). Of the two individuals who reported receiving blood transfusions, the resident of São Benedito reported that this procedure was performed in 1994, while the other did not provide the date on which the transfusion was received. Because clinical screening was established shortly before the woman with HTLV-2 was transfused, there is no certainty as to whether she received screened blood, and thus, the transfusion may have been a source of infection. However, the association with blood transfusion could be a spurious result if we consider the small number of HTLV-1/2 positive subjects found in the present study, which could be considered a limitation of our study.

All three HTLV-2-infected patients have one surname in common; thus, it is possible that there is some degree of kinship between them due to the isolation of these communities. In addition, the 67-year-old woman who was infected reported that she had 11 pregnancies and breastfed all of her children for more than 6 months. However, her children could not be found to be tested, and thus, it is not known whether there was vertical transmission of the virus or if her infection occurred after her pregnancies. A new visit to the community is being arranged to investigate intrafamily transmission.

Although most of those infected with HTLV-1 are asymptomatic, this virus is associated with clinical manifestations that can lead to severe symptoms and death, and there is no cure or treatment available for the infection, which is neglected, not only in Brazil but also in several regions of the world (57). Therefore, HTLV-1/2 infection is a public health problem, especially in vulnerable populations (40, 57).

Although many Brazilian population segments are in a situation of vulnerability, there is a higher prevalence of black populations among the vulnerable populations, including those living in quilombo remnant communities (49, 58). These populations are vulnerable because of health determinants of individual control (behaviors) and collective control (low health conditions and poor infrastructure). Thus, measures such as (i) providing prenatal screening, (ii) providing screening for infection in different quilombola communities, (iii) performing confirmatory tests, (iv) increasing the awareness of the population about the virus and its modes of transmission, (v) encouraging the use of condoms, and (vi) providing counseling for infected pregnant women about the risk of transmission to the child through breastfeeding are means that can mitigate the transmission of HTLV in these communities; these actions promote health promotion/prevention from the perspective of comprehensive care, which is in line with the national policy of comprehensive health for the black population (59).

## Conclusions

Considering the occurrence of HTLV-1/2 infection in populations associated with the African origin of the virus and its introduction into Brazil from the slave trade, the continuous evaluation of quilombola communities in the state of Pará and other locations in the Brazilian Amazon is needed to better characterize the distribution (prevalence) of infection in these populations to better formulate public health policies to control the spread of the virus and associated diseases.

## Data Availability Statement

The raw data supporting the conclusions of this article will be made available by the authors, without undue reservation.

## Ethics Statement

The studies involving human participants were reviewed and approved by Human Research Ethics Committee of the Health Sciences Institute of the Federal University of Pará (CAAE: 55699316.6.0000.0018). The patients/participants provided their written informed consent to participate in this study.

## Author Contributions

AV, JG, ES, IC, and GC-C conceived and designed the study. WB, LR, KP, FL, IA, CL, and AL assisted in subject recruitment and sample collection. BS, FL, WB, IA, and CL performed the serological and molecular analyses. SL and WB performed statistical analyses. WB, GC-C, IC, and AV wrote the manuscript. All authors read and approved the final manuscript.

## Funding

This study was supported by the National Council for Scientific and Technological Development (CNPq; # 301869/2017-0; 442522/2019-3) and the Federal University of Pará(PAPQ-2022).

## Conflict of Interest

The authors declare that the research was conducted in the absence of any commercial or financial relationships that could be construed as a potential conflict of interest.

## Publisher's Note

All claims expressed in this article are solely those of the authors and do not necessarily represent those of their affiliated organizations, or those of the publisher, the editors and the reviewers. Any product that may be evaluated in this article, or claim that may be made by its manufacturer, is not guaranteed or endorsed by the publisher.
